# Supplementary material for: A diminutive new basilosaurid whale reveals the trajectory of the cetacean life histories during the Eocene
Source: Commun Biol. 2023 Aug 10;6:707. doi: 10.1038/s42003-023-04986-w (PMC10415296; doi:10.1038/s42003-023-04986-w)
Supplement: Supplementary file 6 — Supplementary Data 3 [file 42003_2023_4986_MOESM6_ESM.zip › Supplementary Data 3/Supplementary Data 2_BTD_ASR/trait_0032_tree.plot.pdf]

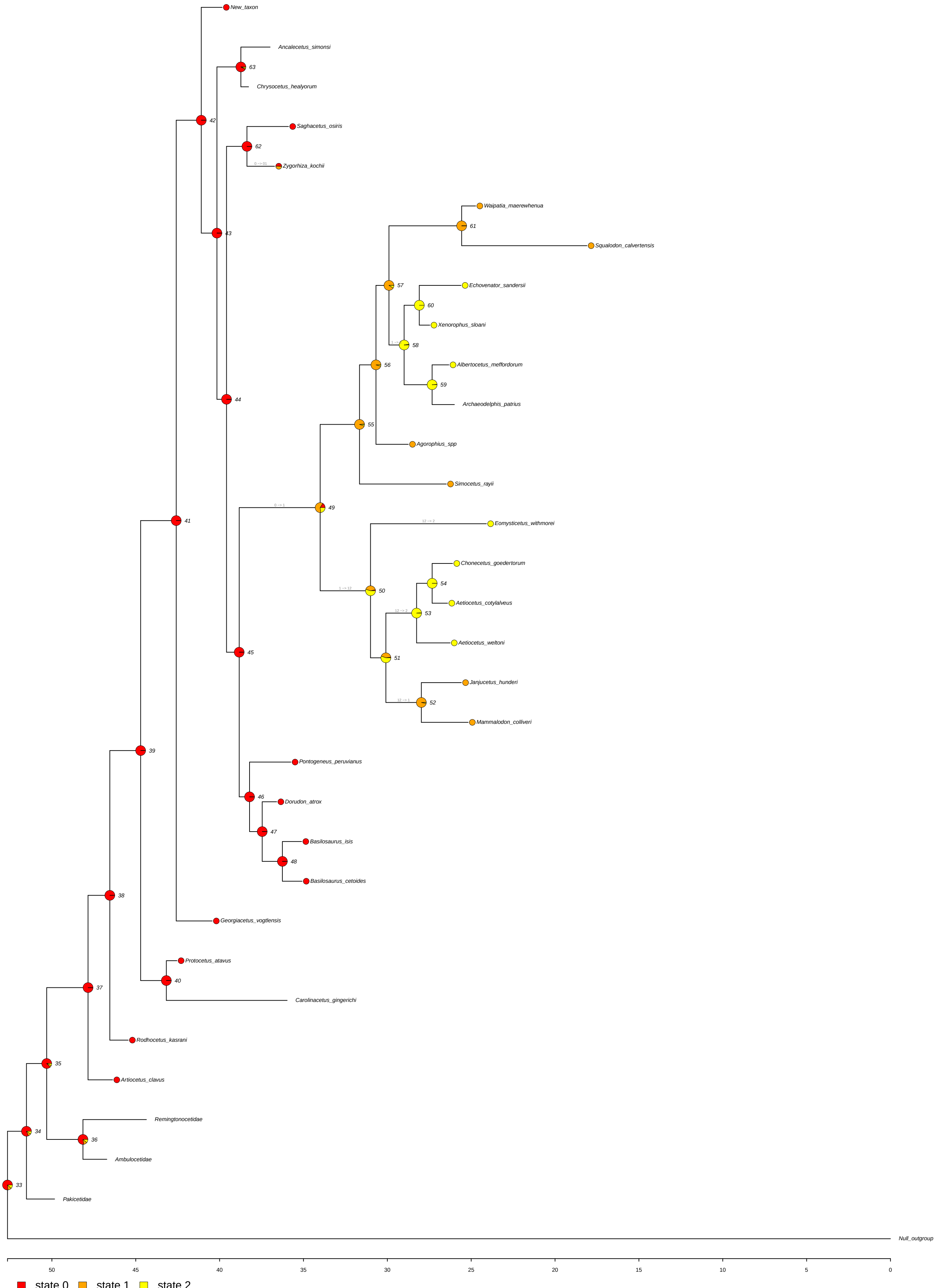

New_taxon
Ancalecetus_simonsi
Chrysocetus_healyorum
Saghacetus_osiris
Zygorhiza_kochii
Waipatia_maerewhenua
Squalodon_calvertensis
Echovenator_sandersii
Xenorophus_sloani
Albertocetus_meffordorum
Archaeodelphis_patrius
Agorophius_spp
Simocetus_rayii
Eomysticetus_withmorei
Chonecetus_goedertorum
Aetiocetus_cotylalveus
Aetiocetus_weltoni
Janjucetus_hunderi
Mammalodon_colliveri
Pontogeneus_peruvianus
Dorudon_atrox
Basilosaurus_isis
Basilosaurus_cetoides
Georgiacetus_vogtlensis
Protocetus_atavus
Carolinacetus_gingerichi
Rodhocetus_kasrani
Artiocetus_clavus
Remingtonocetidae
Ambulocetidae
Pakicetidae
Null_outgroup
33
34
35
36
37
38
39
40
41
42
43
44
45
46
47
48
49
50
51
52
53
54
55
56
57
58
59
60
61
62
63
0 --> 01
0 --> 1
1 --> 12
12 --> 2
12 --> 1
50
45
40
35
30
25
20
15
10
5
0
state 0
state 1
state 2
